# Recent Advances in the Design and Structure–Activity Relationships of Oxygen Evolution Catalysts for Alkaline Water Electrolysis

**DOI:** 10.3390/molecules30224350

**Published:** 2025-11-10

**Authors:** Limin Wang, Xinyue Liu, Cunxiao Lai, Jiabao Liu, Wenqi Wang, Xiaomei Wang, Xin Bo, Tao Cheng, Jianfeng Li, Zenglin Wang, Xubin Lu

**Affiliations:** 1 School of Mechanical Engineering, Lanzhou Jiaotong University, Lanzhou 730070, China; limin.w@163.com; 2 School of Materials Science and Engineering, Lanzhou Jiaotong University, Lanzhou 730070, Chinajiabao.liu2004@outlook.com (J.L.); taocheng9901@163.com (T.C.); 3 Laboratory of Applied Surface and Colloid Chemistry, Ministry of Education, School of Chemistry and Chemical Engineering, Shaanxi Normal University, Xi’an 710062, China

**Keywords:** structure–activity relationships, oxygen evolution reaction, mechanisms, alkaline media, bifunctional catalysts

## Abstract

Electrocatalytic water splitting offers a promising route to sustainable H_2_, but the oxygen evolution reaction (OER) in alkaline media remains the principal bottleneck for activity and durability. This review focuses on alkaline OER and integrates mechanism, kinetics, materials design, and cell-level considerations. Reaction mechanisms are outlined, including the adsorbate evolution mechanism (AEM) and the lattice oxygen mediated mechanism (LOM), together with universal scaling constraints and operando reconstruction of precatalysts into active oxyhydroxides. Strategies for electronic tuning, defect creation, and heterointerface design are linked to measurable kinetics, including iR-corrected overpotential, Tafel slope, charge transfer resistance, and electrochemically active surface area (ECSA). Representative catalyst families are critically evaluated, covering Ir and Ru oxides, Ni-, Fe-, and Co-based compounds, carbon-based materials, and heterostructure systems. Electrolyte engineering is discussed, including control of Fe impurities and cation and anion effects, and gas management at current densities of 100–500 mA·cm^−2^ and higher. Finally, we outline challenges and directions that include operando discrimination between mechanisms and possible crossover between AEM and LOM, strategies to relax scaling relations using dual sites and interfacial water control, and constant potential modeling with explicit solvation and electric fields to enable efficient, scalable alkaline electrolyzers.

## 1. Introduction

The growing global energy demand and reliance on fossil fuels have led to severe environmental issues, including greenhouse gas emissions and resource depletion [1]. To address this, renewable energy sources such as solar, wind, and geothermal power have gained significant attention. However, their intermittent nature and limited efficiency in energy conversion and transmission limit widespread adoption [2,3]. Hydrogen is a clean and efficient energy carrier that presents a promising solution by allowing renewable energy to be stored and utilized through water electrolysis [4,5,6,7]. In this review, we emphasize alkaline oxygen evolution reaction (OER) as the principal kinetic bottleneck in water electrolysis and center the discussion on OER-specific mechanisms, descriptors, and design principles.

Water electrolysis comprises two half-reactions that proceed concurrently. In principle, it can approach the thermodynamic minimum cell voltage of about 1.23 V at 25 °C with near-unity Faradaic efficiency [8,9]. However, in alkaline media, the sluggish OER kinetics impose overpotential that raises the required cell voltage, in addition to ohmic and mass transport losses, making the development of active and durable OER electrocatalysts a critical research focus [10,11,12].

Recent advancements in electrocatalyst development have shifted from using expensive noble metals like platinum, ruthenium, and iridium to more abundant transition metal-based alternatives. These include oxides, nitrides, and carbon-based materials, with strategies such as defect engineering, heterostructure formation, and surface modifications enhancing their performance [13,14,15].

Accordingly, this review concentrates on OER. It outlines electrochemical parameters that govern water splitting and provides a concise overview of widely accepted alkaline OER mechanisms, including their reaction pathways. It then examines how structural features influence catalytic performance and highlights strategies for achieving both high activity and long-term stability. It also categorizes major classes of alkaline OER catalysts and summarizes their design principles and structure–activity relationships. Finally, it discusses key challenges and future research directions for water electrolysis in hydrogen production.

## 2. Electrocatalytic Water Splitting

**Figure 1:**
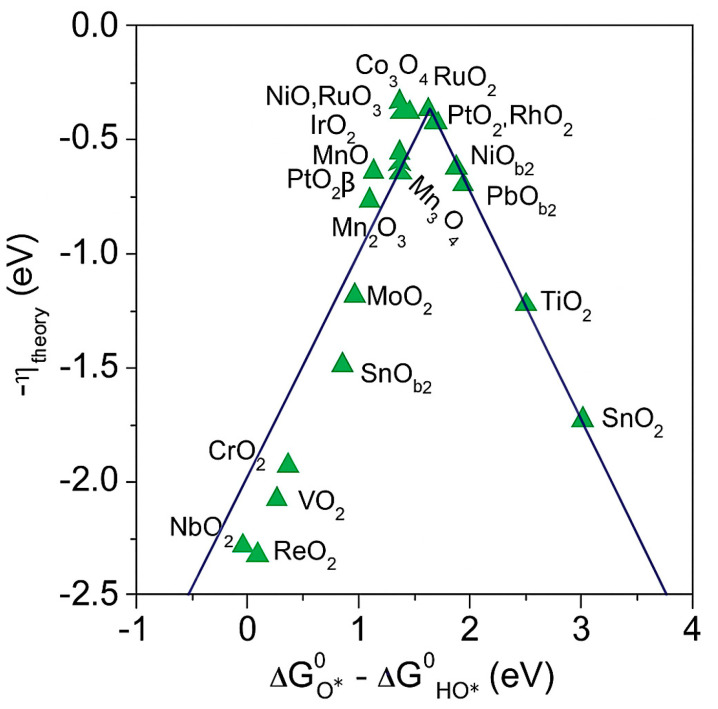
Volcano curve of OER [22]. Reproduced from Ref. [22] with permission. Copyright 2021 Wiley.

The volcano-shaped curve in Figure 1 illustrates the catalytic performance across materials for HER and OER. Researchers have long sought catalysts that are highly active, stable, and cost-effective. Current research primarily focuses on two approaches. The first approach is the discovery and development of efficient non-precious metal catalysts and their compounds, which are abundant, affordable, and sufficiently active, providing practical alternatives to precious metals and lowering both cost and supply risk. The second approach is the optimization of precious metal catalysts and their supports to reduce noble metal usage while improving durability, thereby raising overall cost effectiveness. While precious metals such as Pt and Ir are still widely used, their expense and durability constraints continue to motivate substitution strategies and hybrid designs. Researchers are exploring cobalt, nickel, and molybdenum systems together with coordination polymers, advanced carbon scaffolds, and engineered supports.

Additionally, alkaline or near-neutral electrolytes are generally preferred for industrial-scale water electrolysis. Acidic environments are typically corrosive to many metals, reducing catalyst lifetime and raising balance-of-plant concerns. Alkaline or near-neutral conditions tend to stabilize oxide surfaces and favor hydroxide-mediated OER pathways.

### 2.1. Oxygen Evolution Reaction

OER is the anodic half-reaction in water electrolysis. It requires four electron transfers and the formation of an O–O bond. In both acidic and alkaline media, the reaction proceeds through surface intermediates OH*, O*, and OOH*. In an acidic electrolyte, protons come from the electrolyte, and water supplies oxygen to the intermediates [16,17,18]. In an alkaline electrolyte, hydroxide adsorbs and deprotonates on the surface to generate the same intermediates, and the electrolyte serves as the proton acceptor. Differences in donors, acceptors, and surface coverage shift the rate-determining step and the observed kinetics.

The efficiency and stability of OER catalysts are largely set by the rate-determining step, which is the step with the highest activation barrier [19]. According to the Sabatier principle, optimal activity occurs when intermediates bind neither too strongly nor too weakly, which provides a useful framework to interpret mechanisms and kinetics [20]. Excessively strong binding traps intermediates and hinders O_2_ release, whereas overly weak binding leads to premature desorption and slower rates [21].

Acidic:

H_2_O(l) + * → OH* + H^+^ + e^−^

OH* → O* + H^+^ + e^−^

O* + H_2_O(l) → OOH* + H^+^ + e^−^

OOH* → * + O_2_(g) + H^+^ + e^−^

In an alkaline electrolyte, OH^−^ participates in forming the same types of intermediates.

Alkaline:

OH^−^ + * → OH* + e^−^

OH* + OH^−^ → O* + H_2_O + e^−^

O* + OH^−^ → OOH* + e^−^

OOH* + OH^−^ → * + O_2_(g) + H_2_O(l) + e^−^

A common thermodynamic descriptor is the adsorption free-energy difference ΔG_O*_ − ΔG_OH*_, where ΔG_*O_ and ΔG_OH*_ are the free energies of O* and OH* on the surface (* denotes an adsorption site). This difference correlates with the electronic structure and the surface crystal structure, and it can be used to predict OER activity across materials [23]. As shown in Figure 1, the theoretical overpotential follows a volcano relation with ΔG_O*_ − ΔG_OH*_. IrO_2_, RuO_2_, NiO_x_, and Co_3_O_4_ lie near the apex, which indicates near-optimal binding and high activity. CrO_2_, VO_2_, SnO_2_, TiO_2_, and ReO_2_ tend to lie on the flanks of the volcano and, therefore, show lower activity due to non-optimal adsorption energetics, which may arise from either overly strong or overly weak binding depending on the side of the apex.

It is important to note that ΔG_O*_ − ΔG_OH*_ is a thermodynamic descriptor. Actual rates also depend on activation barriers, on potential-dependent coverage, transport, and electrolyte composition. Many materials are reconstructed under operating potentials to active oxyhydroxide phases, which complicates mechanism assignment and activity evaluation. Thermodynamic descriptors should, therefore, be combined with kinetic parameters and operando characterization.

### 2.2. Electrochemical Parameters of Water Electrolysis

Volcano plots offer a first look at catalytic activity, but a complete performance assessment requires electrochemical metrics that can be measured and fitted, including the Tafel slope, the overpotential at a defined current density, and impedance-derived charge transfer resistance. These parameters report how reaction intermediates interact with the catalyst surface and how efficiently electrons move across the interface [24]. Comparing them across materials reveals structure–activity trends and guides the design and optimization of more effective catalysts. Combining volcano curve analysis with quantitative electrochemical characterization is now standard for evaluating catalyst performance. Experimental and theoretical tools used together can then identify active motifs and stability limits, enabling the rational development of next-generation catalysts for broader applications.

Overpotential

**Figure 2:**
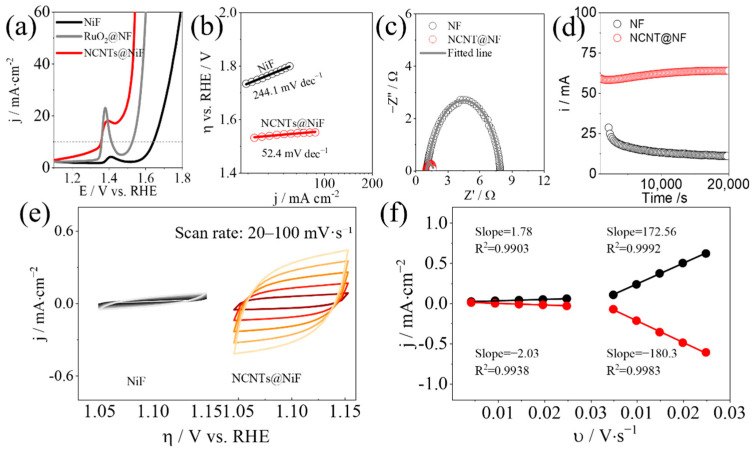
Electrocatalytic properties of NiF and NCNTs@NiF for OER in 1 M KOH. (**a**) LSV; (**b**) Tafel slopes; (**c**) EIS; (**d**) stability at 1.58 V vs. RHE; (**e**,**f**) non-Faradaic CVs and slopes.

In electrocatalytic and photoelectrocatalytic systems, the operating voltage often exceeds the thermodynamic value. This excess is the overpotential. Kinetic barriers make the working voltage higher than the equilibrium potential. Overpotential (η) reflects activation resistance, charge transfer resistance, and other losses and, therefore, tracks catalytic activity [25]. A smaller overpotential indicates lower energy consumption and higher efficiency. Comparisons must be made at a defined current density, for example, 10 mA·cm^−2^. Linear sweeping voltammetry (LSV) is recorded versus the reversible hydrogen electrode (RHE). As shown in Figure 2a, nitrogen-doped carbon nanotubes on nickel foam (NCNTs@NiF) deliver the lowest overpotential among the tested catalysts.

Tafel slope

The Tafel slope evaluates apparent kinetics and can be obtained from LSV after appropriate iR-corrected compensation. The Tafel relation is η = a + b log j, where η is the overpotential and j is the current density [26]. Smaller b indicates faster apparent kinetics. In Figure 2b, NCNTs@NiF exhibits the lowest Tafel slope.

Charge transfer resistance

Charge transfer processes significantly influence the catalytic activity of a material. The lower the charge transfer resistance (R_ct_), the easier it is for electrons to transfer across the catalyst surface, enhancing the overall catalytic performance. R_ct_ values are typically obtained using electrochemical impedance spectroscopy (EIS) [26]. The resulting EIS plots usually form a semicircular shape, with the diameter representing the R_ct_ value. A smaller semicircle diameter indicates a lower R_ct_ value, which correlates to better charge transfer capabilities and higher catalytic activity. NCNTs@NiF has the smallest semicircle, corresponding to the lowest R_ct_ value and the best catalytic activity. Measuring R_ct_ provides an objective assessment of the speed and efficiency of charge transfer on the catalyst surface, offering valuable insights for catalyst optimization, as shown in Figure 2c.

Long-term stability

Catalyst stability describes whether performance remains constant or varies only slightly during repeated operation. Stability is a critical criterion for evaluating overall catalytic performance [27]. Stability can be assessed by quantifying activity loss during operation, comparing surface structure before and after testing, and evaluating resistance to environmental stressors such as water exposure. Changes in surface chemistry or crystal structure can destroy active sites and diminish catalytic activity, as shown in Figure 2d.

Electrochemically active surface area

The ECSA was estimated from double-layer capacitance (C_dl_) using scan-rate-dependent cyclic voltammetry in a non-Faradaic window [26]. Under these conditions, the measured current arises primarily from double-layer charging and scales linearly with the ECSA. C_dl_ was obtained from linear fits of current versus scan rate by averaging the absolute anodic and cathodic slopes, as shown in Figure 2e,f.

$$C_{dl\;}=\frac{|\mathrm{Slope}\left(\mathrm{anode}\right)|+|\mathrm{Slope}\left(\mathrm{cathode}\right)|\;}{2}$$

NiF served as the substrate and reference. The relative ECSA of each catalyst was computed by dividing its C_dl_ by the C_dl_ of pristine NiF, giving

$${ECSA}_{rel}=\frac{{\;C_{dl\;}catalyst\;(mF\cdot cm}^{-2\;})}{C_{dl}\;Ni\;foam\;\left({mF\cdot cm}^{-2}\right)\;per\;ECSA\;{cm}^{2}}$$

As an example under OER conditions, the relative ECSA of NCNTs@NiF is obtained by applying this ratio to the measured C_dl_ values for NCNTs@NiF and NiF.

$$C_{dl}(NiF)=\frac{\left|1.78|+\right|-2.03|\;}{2}=1.91\;mF\cdot cm^{-2}$$

$$C_{dl}(NCNTs)=\frac{\left|172.56\right|+\left|-180.3\right|}{2}=176.43\;mF\cdot cm^{-2}$$

$$A_{\mathrm{ECSA}}^{\mathrm{NCNTs}}=\frac{{\;176.43\;\mathrm{mF}\cdot \mathrm{cm}}^{-2\;}}{1.91\;{\mathrm{mF}\cdot \mathrm{cm}}^{-2}\;\mathrm{per}\;\mathrm{ECSA}\;{\mathrm{cm}}^{2}}=92.4{cm}_{ECSA}^{2}\;$$

Turnover frequency

TOF reports the intrinsic rate per active site, defined as the number of O_2_ molecules formed per site per second. For OER, the four-electron stoichiometry gives the instantaneous O_2_ formation rate from the measured current [28].

$$r_{O_{2\;}\;}=\mathrm{iFE}(\frac{N_{A}\;}{\mathrm{zF}}),\;\text{with}\;z=4,$$

where i is the steady Faradaic current, FE is the Faradaic efficiency, N_A_ is Avogadro’s number, z = 4 for OER, and F is the Faraday constant. TOF then follows as

$$\mathrm{TOF}\;=\frac{r_{O_{2\;}\;}\;}{N_{\mathrm{sites}}}\;(S^{-1}),$$

with N_sites_ being the number of electrochemically addressable active sites.

An equivalent area-normalized form uses current density j and the areal density of active sites Γ_act_ in mol·cm^−2^

$$\mathrm{TOF}\;=\frac{\mathrm{jFE}\;}{{zF\Gamma }_{\mathrm{sites}}},\;\text{with}\;z=4.$$

Active-site counts are typically obtained from integrated redox charge of surface metal centers (for example, Ni^2+^/Ni^3+^ or Fe^3+^/Fe^4+^) or from independent estimates of surface metal dispersion.

## 3. Oxygen Evolution Reaction Mechanisms

**Figure 3:**
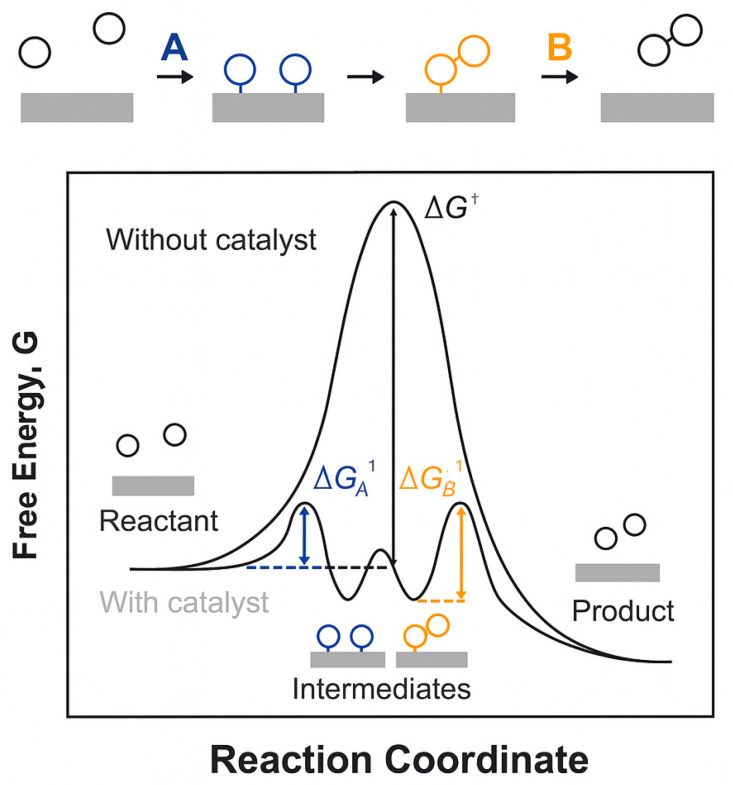
Schematic representation of the typical reaction mechanism with and without a catalyst during the OER [29]. ΔG^†^ denotes the activation free energy barrier. Reproduced from Ref. [29] with permission. Copyright 2015 RSC Publishing.

The performance of OER catalysts directly influences the efficiency and cost of hydrogen production via water electrolysis. Therefore, a deep understanding of OER mechanisms is essential. Catalysts modify reaction pathways by stabilizing key intermediates and transition states, which lowers activation barriers and accelerates the overall rate [29]. Figure 3 presents a representative energy profile and illustrates how catalysts reduce barriers for surface intermediates and create lower-energy transition states. This indicates that the catalyst interacts with reactants at multiple stages to decrease activation energies and increase rates.

The OER pathway is complex and typically exhibits slow kinetics, which makes it the most demanding step in electrocatalytic water splitting [30]. In alkaline media, OER primarily involves oxidation of OH^−^. Electron transfer kinetics are intricate, and the exact mechanism remains under debate. Two widely discussed frameworks are the adsorbate evolution mechanism (AEM) and the lattice oxygen-mediated mechanism (LOM) [31,32]. Additional proposals include proton acceptor and dual-site cooperative pathways [33]. As experimental and computational techniques improve, further mechanisms will likely be proposed and validated, which should sharpen mechanistic assignments under operating conditions.

### 3.1. Adsorbate Evolution Mechanism

In OER under aqueous conditions, the relevant species that approach the surface are water and hydroxide. Molecular O_2_ is formed on the surface during turnover and is not a reactant. AEM describes a sequence in which these species form oxygenated adsorbates that subsequently couple to generate O_2_. Classical adsorption theory provides useful language for describing these steps. Diffusion brings reactants from the electrolyte to the interface. Adsorption places them on surface sites with an associated adsorption free energy. Activation reorganizes electrons and nuclei to form new bonds that define the next intermediate [34]. Foundational models, such as the Langmuir and the Freundlich isotherms, originally developed for gas–solid systems, have since been generalized to liquid–solid interfaces and inform analysis of coverage and site occupancy on electrodes [35]. These ideas underpin contemporary descriptions of adsorbate-controlled electrocatalysis in water splitting [36,37].

Around 1920, Taylor recognized that defects such as steps, corners, and kinks serve as highly active centers, which focused attention on surface heterogeneity and site specificity [38]. Contemporary surface science links site-specific structure to measurable kinetics, providing a consistent basis for OER design. Furthermore, the enthalpy of intermediate formation directly tunes stability and turnover [39]. Consequently, volcano relations that correlate activity with thermodynamic descriptors, such as ΔG_O*_ − ΔG_OH*_, capture how energetics map onto rates [40,41]. Therefore, AEM assumes a limited set of sites and a largely static surface, which can miss heterogeneity, dynamic reconstruction, and site pairing. Whether AEM or LOM dominates depends on the electronic structure and the availability of single or paired sites. Well-ordered oxides with few defects often favor AEM, proceeding through *OOH on single sites or through O–O coupling on adjacent sites [40]. Amorphous oxides and perovskites with abundant oxygen vacancies and high metal–oxygen covalency frequently show LOM signatures, where lattice oxygen participates directly. This participation can increase activity but may compromise stability by creating vacancies that require replenishment and by exposing unsaturated metal centers that are susceptible to dissolution [42,43].

### 3.2. Lattice Oxygen-Mediated Mechanism

LOM involves the continual release and replenishment of lattice oxygen during OER. As oxygen content fluctuates, the thermodynamic properties of the catalyst surface change accordingly. LOM requires prior activation of lattice oxygen. Once activated, lattice oxygen enters the catalytic cycle, interacts with vacancies, and participates in subsequent turnovers [44,45].

Lattice oxygen participation is favored when metal-d and oxygen 2p states strongly hybridize. When the oxygen 2p band approaches or crosses the Fermi level relative to the metal-d band, electron density can transfer from O-2p to metal-d. This process creates a ligand hole character on oxygen, often denoted O^−^. Neighboring O^−^ species can couple to form [O_2_]^n−^ units, which initiates lattice oxygen involvement in OER [45]. During turnover, activated lattice oxygen typically reacts at dual metal sites where OH first adsorbs and deprotonates to O species, and adjacent O species then couple to form an O–O bond, release O_2_, and begin a new lattice oxygen cycle.

**Figure 4:**
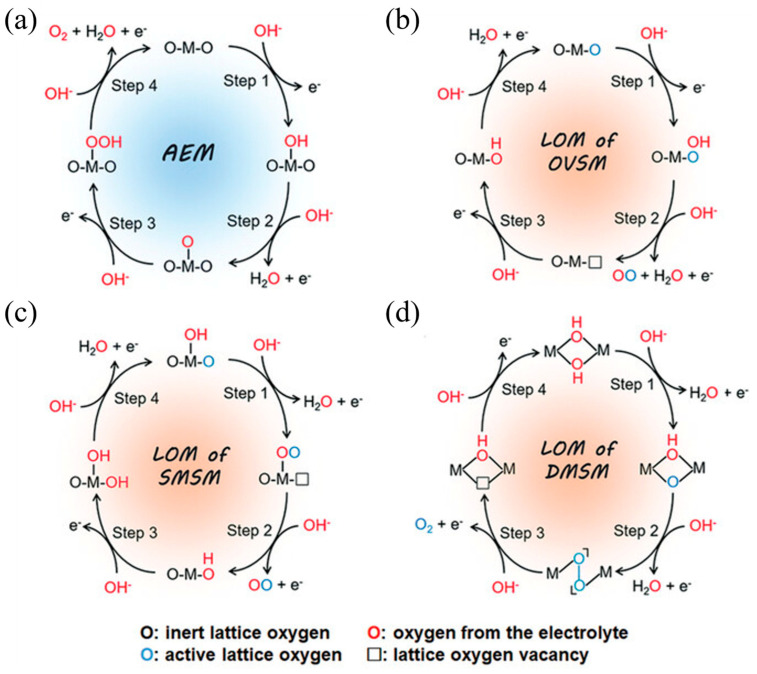
The schematic illustration of AEM and LOM pathways for alkaline OER [46]. (**a**) AEM pathway on an active metal site. (**b**) LOM via an oxygen-vacancy site mechanism (OVSM). (**c**) LOM via a single-metal-site mechanism (SMSM). (**d**) LOM via a dual-metal-site mechanism (DMSM). Reproduced from Ref. [46] with permission. Copyright 2023 Wiley.

Three classes of active centers are commonly considered: an oxygen vacancy pathway, a single metal site pathway, and a dual metal site pathway [43,44]. In the oxygen vacancy pathway, the reaction proceeds via a nucleophilic attack, leading to the formation of an OOH* intermediate. Subsequently, an O_2_ molecule is released, leaving behind a lattice oxygen vacancy. This vacancy is then filled by an OH* species in the subsequent catalytic cycle. In the dual metal site pathway, two adjacent activated lattice oxygen atoms couple to form an M–OO–M motif. O_2_ evolves, two vacancies form, and both are refilled by OH to close the cycle. In the single metal site pathway, one metal atom adsorbs OH, deprotonates to an O species, forms an O–O intermediate, and releases O_2_, and the generated vacancy is refilled by OH, as shown in Figure 4a–d.

**Figure 5:**
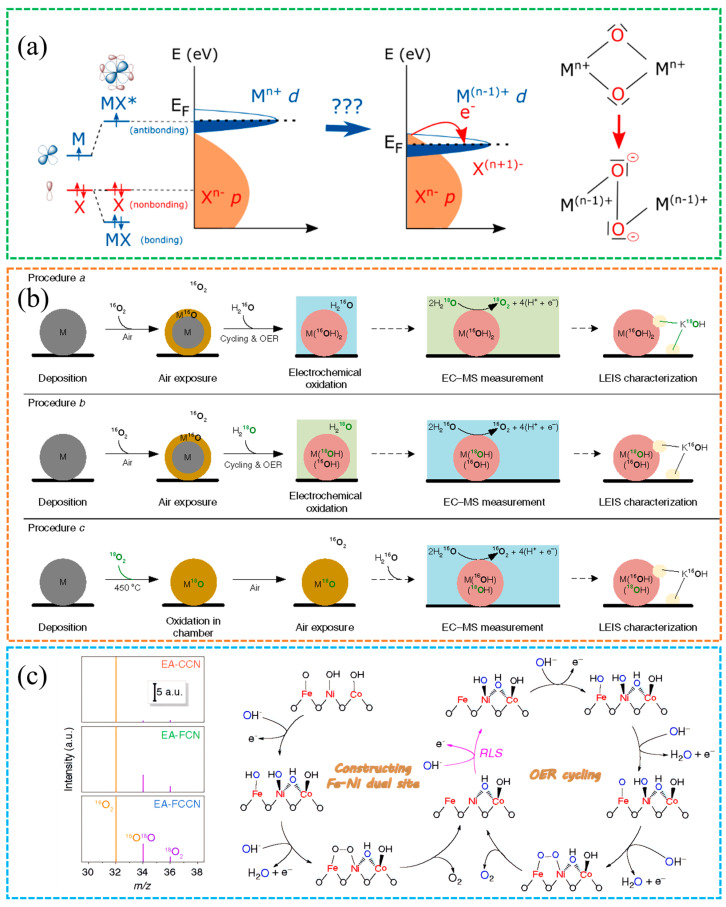
(**a**) Changes in the energy bands near the Fermi level due to lattice oxygen participation [47]. Reproduced from Ref. [47] with permission. Copyright 2016 Springer Nature. (**b**) In situ ^18^O isotope labeling and mass spectrometry confirm the LOM mechanism [49]. Reproduced from Ref. [49] with permission. Copyright 2018 Springer Nature. (**c**) Schematic of ^18^O-labeled OER pathway [28]. Reproduced from Ref. [28] with permission. Copyright 2020 Springer Nature.

A defining feature of the LOM mechanism is the direct participation of lattice oxygen in O–O bond formation, which links neighboring sites and enables surface oxygen exchange that can increase catalytic efficiency. This behavior emerges when O 2p and metal-d states strongly hybridize so that oxygen develops a ligand hole character near the Fermi level, providing an electronic pathway for O-centered charge transfer and peroxo-like motifs under polarization [47]. Operationally, three classes of reaction centers are often discussed: an oxygen vacancy route in which O_2_ evolution leaves a vacancy that hydroxide refills in the next cycle, a single metal route where one metal site hosts adsorption, deprotonation, O–O formation and release, and a dual metal route where adjacent lattice oxygens couple to form an M–OO–M intermediate before O_2_ evolution. Defect-rich NiFe oxyhydroxides and perovskite-like oxides with high metal–oxygen covalency frequently exhibit these signatures, and Fe sites embedded in the NiOOH lattice have been pinpointed as catalytically dominant during OER, clarifying site identity and electronic contributions [48], whereas well-ordered oxides with fewer vacancies tend to favor adsorbate-controlled sequences, as shown in Figure 5a.

Figure 5b shows that evidence from complementary material systems supports this picture. Chorkendorff et al. reported that nanoparticle size and the availability of lattice oxygen jointly influence water oxidation on NiFeO_x_H_y_, and isotope labeling confirms incorporation of lattice oxygen into evolved O_2_, consistent with a lattice exchange route [49]. Chai et al. [28] showed that high-valence metal sites enhance O-2p metal-d hybridization, which lowers the barrier for O–O bond formation and increases water oxidation rates, as shown in Figure 5c. Spectroscopic trends that evolve with potential, for example, features in the O-K edge and metal L-edges, offer practical diagnostics for tracking oxygen redox and linking it to catalytic behavior. Taken together, LOM better captures surface heterogeneity and multiple centers and naturally accounts for lattice oxygen participation. Operando spectroscopies with density functional theory (DFT) deepen insights [50,51,52]. These additional mechanisms still require rigorous validation under operating conditions [33].

LOM becomes advantageous when oxygen vacancy healing is fast and cation dissolution is minimal under load. Materials with O-2p states near the Fermi level and moderate vacancy formation energies tend to benefit. Shao et al. [53] showed at approximately 50–80 °C that heating increases lattice oxygen participation and apparent OER rates by enhancing vacancy mobility and O-2p to metal-d hybridization while also accelerating A-site and B-site leaching and phase drift. Thus, pair fast oxygen exchange with robust vacancy re-oxidation and cation retention; validate with ≥100 mA·cm^−2^, small η drift, consistent Tafel slopes, and low R_ct_.

## 4. Rational Design of Oxygen Evolution Catalysts

**Figure 6:**
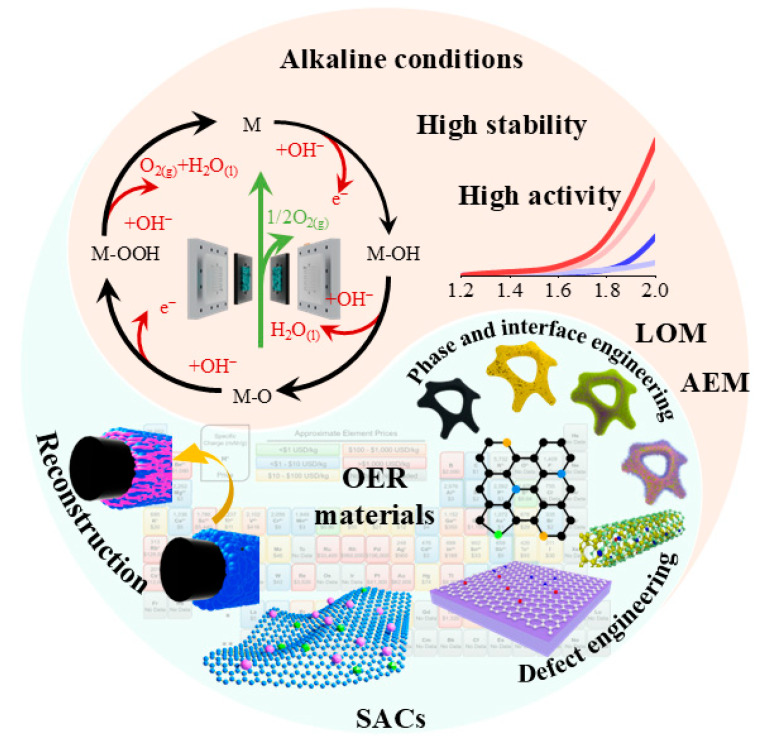
Summarizes design levers and strategies for alkaline OER.

Lowering the cost of renewable hydrogen depends on OER catalysts that combine high activity with durability. Meeting this goal requires deeper insight into kinetic barriers and atomic-scale structure–activity relationships to guide rational design, as shown in Figure 6.

In practice, the rate-determining step sets the most useful guide for materials design and synthesis. Building on the AEM and LOM frameworks introduced earlier and supported by theory that evaluates barriers and free energies for key intermediates, rational design aims to lower kinetic hurdles while stabilizing the working state of the catalyst. Adjusting the relative energetics of OH*, O*, and OOH* is essential for reducing the theoretical overpotential. More precisely, relaxing the universal scaling relations among these intermediates is the credible route to approach very low overpotentials rather than to reach zero.

IrO_2_ and RuO_2_ remain reliable choices for minimizing overpotential and ensuring durability, yet reliance on precious metals limits scalability. Non-precious catalysts in alkaline media still face gaps in intrinsic activity and stability, although advances in composition and structure are narrowing those gaps. Two-dimensional non-precious systems already show promise, where heteroatom incorporation, hierarchical porosity, and conductive supports improve electronic structure, transport, and gas release.

Current design strategies center on five levers that can be combined. Phase and interface engineering tunes crystal structure and short-range order to stabilize active phases under bias. Single-atom engineering maximizes metal utilization and tailors the local coordination to optimize adsorption thermodynamics. Dual single-atom motifs introduce neighboring cooperative sites that can weaken single-site scaling limits and facilitate O–O bond formation. Defect engineering uses controlled vacancies, strain, and coordination disorder to modulate electronic structure and the electrolyte interface. Reconstruction engineering exploits in situ transformation to oxyhydroxides or interfacial phases so that the operando state aligns with the most active configuration. Together, these approaches target the rate-determining step, preserve the active phase during long operation, and move alkaline OER toward lower overpotentials with longer lifetimes.

### 4.1. Reconstruction Engineering

In alkaline OER, most transition metal precatalysts do not operate in their as-synthesized state. Rather, they undergo in situ transformation to thin surface (oxy)hydroxide skins whose composition, thickness, and defect chemistry ultimately govern both activity and lifetime. Reconstruction engineering, therefore, centers on three coupled levers: triggering and steering formation of the active (oxy)hydroxide under bias, coupling that formation to fast charge and mass transport at realistic current densities, and stabilizing the reconstructed phase against metal dissolution and corrosive species.

**Figure 7:**
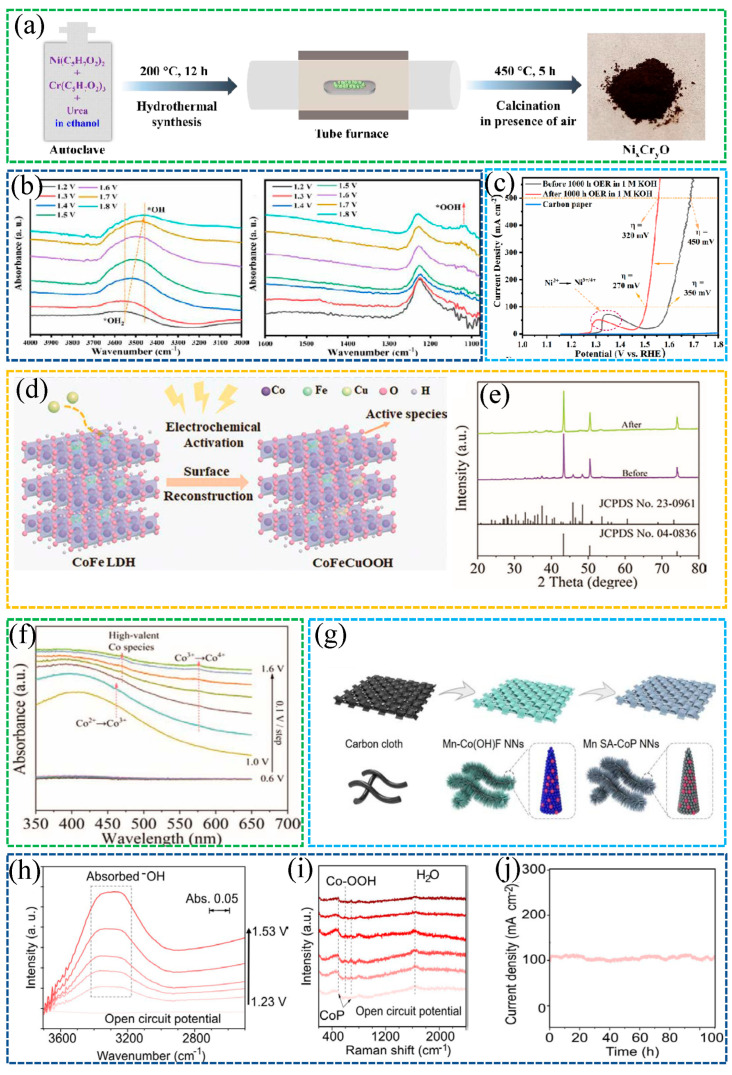
(**a**) Synthesis scheme of Ni_x_Cr_γ_O. (**b**) Operando ATR-IR showing potential-dependent *OH and *OOH bands during OER. (**c**) LSV before and after OER in 1 M KOH [54]. Reproduced from Ref. [54] with permission. Copyright 2023 Wiley. (**d**) Schematic of surface reconstruction from CoFe LDH to CoFeCuOOH. (**e**) XRD patterns before and after reconstruction. (**f**) Operando UV–vis spectra of Cu_2_S/CoFe LDH from open-circuit to 1.6 V vs. RHE [57]. Reproduced from Ref. [57] with permission. Copyright 2022 Wiley. (**g**) Synthesis route of Mn SA-CoP nanoneedles on carbon cloth. (**h**) In situ ATR-IR of adsorbed *OH on Mn SA-CoP NNs at increasing potentials. (**i**) Operando Raman spectra of Mn SA-CoP NNs. (**j**) Chronoamperometric stability of Mn SA-CoP NNs in alkaline OER [59]. Reproduced from Ref. [59] with permission. Copyright 2024 Wiley.

Malek et al. [54] developed a NixCryO electrocatalyst that manifested exceptional OER stability in alkaline conditions while improving the activity over time by dynamic self-restructuring, as shown in Figure 7a,b. The catalyst forms an in situ Ni/Cr (oxy)hydroxide skin under anodic bias. Mechanistically, Cr incorporation moderates surface electronic structure, suppresses Cl^−^ attack and metal dissolution, and helps lock the reconstructed (oxy)hydroxide phase, while maintaining rapid OH^−^ adsorption/charge transfer. In 1 M KOH, Ni_x_Cr_y_O required overpotentials of only 270 mV and 320 mV to achieve current densities of 100 and 500 mA cm^−2^, respectively, with excellent long-term stability, as shown in Figure 7c.

Aalling-Frederiksen et al. [55] tracked NiFe-LDH during OER and observed rapid conversion to β/γ-NiOOH with Fe incorporation at working potentials. The degree of reconstruction and changes in metal–oxygen covalency/Fe valence correlated with lower overpotential and Tafel slope. Linke et al. [56] used a nickel metal–organic framework 74 (Ni-MOF-74) as a precursor and, via operando/near-operando spectroscopy (XAS/Raman) and electrochemistry, tracked its in situ reconstruction into a Ni(oxy)hydroxide (β/γ-NiOOH-like) working skin under alkaline bias. The transformation correlated with lower η at j, smaller Tafel slopes, and reduced EIS versus the pristine MOF, indicating faster interfacial kinetics once the reconstructed phase formed. Long-term polarization further showed a stabilized reconstructed layer with suppressed metal dissolution, delivering durable OER in alkaline media and device-relevant performance on conductive supports. Operando evidence, therefore, supports Ni and Fe containing (oxy)hydroxides as the working state and enables identification of the active motifs and operative pathways during reconstruction.

Wang et al. [57] electrodeposited a CoFe layered double hydroxide (CoFe LDH) precatalyst onto a Cu_2_S scaffold (Cu_2_S/CoFe LDH) and, upon electrochemical activation, obtained the reconstructed CoFeCuOOH, as shown in Figure 7d. The resulting CoFeCuOOH delivers excellent alkaline OER performance, requiring only ~170 mV overpotential to reach 10 mA·cm^−2^ in 1 M KOH. As shown in Figure 7e, ex situ XRD before and after activation confirms the phase evolution consistent with surface reconstruction. As shown in Figure 7f, operando UV–vis spectra track the potential-dependent growth of high-valent Co features, evidencing irreversible transformation of the LDH on Cu_2_S into a high-valent, Co-rich (oxy)hydroxide working state. Density of states analysis further indicates intramolecular electron redistribution and ligand hole formation that facilitate active-site reconstruction. In situ ^18^O-labeling differential electrochemical mass spectrometry, together with DFT, identifies lattice oxygen-based active sites and supports an oxygen vacancy site pathway.

Sun et al. [58] tracked the structural evolution of Ni-based precatalysts, including Ni(OH)_2_, NiS_2_, NiSe_2_, and NiTe, using operando spectroscopy coupled with theory to identify their true active states during water oxidation. They revealed sub-nanometric differences in the reconstructed NiOOH phase. The NiO_6_ octahedron adopts a regular yet distinct distortion whose magnitude is jointly governed by the geometric bond length and electronic covalency characteristics of the precatalyst across space and time. This symmetry-broken NiO_6_ unit rebalances O-2p and Ni-3d orbital interactions, steers intermediate configurations and operative pathways, and enhances OER performance.

With the working state established, accelerating its formation is an effective route to raise apparent kinetics. Shi et al. [60] implemented reconstruction rate engineering to shorten the LDH to β-NiOOH transition, which lowered overpotential and Tafel slope at matched loading and electrolytes. This result provides direct evidence that shortening the induction period to the active phase improves rate metrics.

Wang et al. [59] designed Mn single-atom-doped CoP nanoneedles (Mn SA–CoP NNs) that were reconstructed in situ under anodic bias to a thin Co(oxy)hydroxide working skin. The fabrication route of Mn SA–CoP NNs is shown in Figure 7g. Operando ATR-IR in Figure 7h and Raman in Figure 7i revealed strengthened *OH adsorption and the emergence of Co-OOH features, evidencing surface reconstruction. Mn single atoms acted as atomically localized electric field centers, while the needle geometry provided nanoscale field confinement. Together, they enriched interfacial OH^−^ and facilitated *O/*OOH desorption, accelerating the OER cycle. In alkaline tests, the catalyst achieved an overpotential of ≈189 mV at 10 mA·cm^−2^ and maintained stable operation for ~100 h at ≈100 mA·cm^−2^, as shown in Figure 7j. It also exhibited a lower Tafel slope, EIS, and a higher ECSA than undoped CoP.

Zhu et al. [61] investigated CuS versus Cu_2_S as alkaline OER precursors under anodic bias. CuS undergoes a stepwise transformation in which S^2−^ is oxidized to SO_4_^2−^ and Cu^+^ converts to CuO via a Cu (OH)_2_ intermediate, forming a thin working (oxy)hydroxide or oxide skin. Operando and near-operando characterization link the extent of reconstruction to improved kinetics. A modified hourglass framework in CuS strengthens Cu–S bonding and improves conductivity, which accelerates electron transport and facilitates *OH, *O, and *OOH formation relative to Cu_2_S.

Liu et al. [62] developed an ion migration-mediated stabilization route by cathodic polarization of Fe-rich NiFe-LDH to obtain R-NiFe-LDH, which drives Ni → Fe cation exchange and oxyanion → OH^−^ substitution, yielding γ-(NiFe)OOH with suppressed Fe dissolution. R-NiFe-LDH sustains 500 mA·cm^−2^ with ~90% Fe retention over 5 days and shows only ~70 mV overpotential rise over two months. In zero-gap AEM tests, using R-NiFe-LDH as both electrodes yields no measurable decay at 1000 mA·cm^−2^ over 100 h.

Therefore, these results delineate a coherent, mechanism-anchored toolkit: verify and exploit the reconstructed (oxy)hydroxide as the working phase using operando identification, accelerate its formation through reconstruction rate control, steer and assist reconstruction with dopants, heterointerfaces, and local electric field microenvironments, and stabilize the reconstructed state through compositionally guided approaches. Across this toolkit, claims should be benchmarked under device-relevant conditions, including iR-corrected overpotential at current densities of at least 100 to 500 mA·cm^−2^, Tafel analysis, EIS and ECSA or TOF, metal loss metrics, and zero-gap anion exchange membrane cell voltages, so that gains in activity and durability are demonstrated concurrently.

### 4.2. Defect and Vacancy Engineering

Defect engineering enhances electrocatalytic activity by introducing structural defects, such as vacancies, grain boundaries, and strains, or chemical defects, such as heteroatom doping into catalyst materials. This strategy tailors the electronic structures, surface active sites, and adsorption/desorption properties of the catalysts to achieve activity enhancement [63,64].

Vacancy defects are vacant sites formed by the absence of atoms in the crystal lattice. These vacancies can alter the local electron density of the catalyst and create an unsaturated coordination environment. They not only provide new adsorption sites for reactants but also optimize the adsorption energy of reaction intermediates, ultimately boosting catalytic performance. Furthermore, atomic vacancies can be intentionally introduced into MXene materials. MXene nanomaterials exhibit tremendous potential in electrocatalytic water splitting, particularly for HER and OER, owing to their distinctive physicochemical properties. Notably, the introduction of atomic vacancies facilitates a further boost in the catalytic activity of the MXene-based catalyst [65].

**Figure 8:**
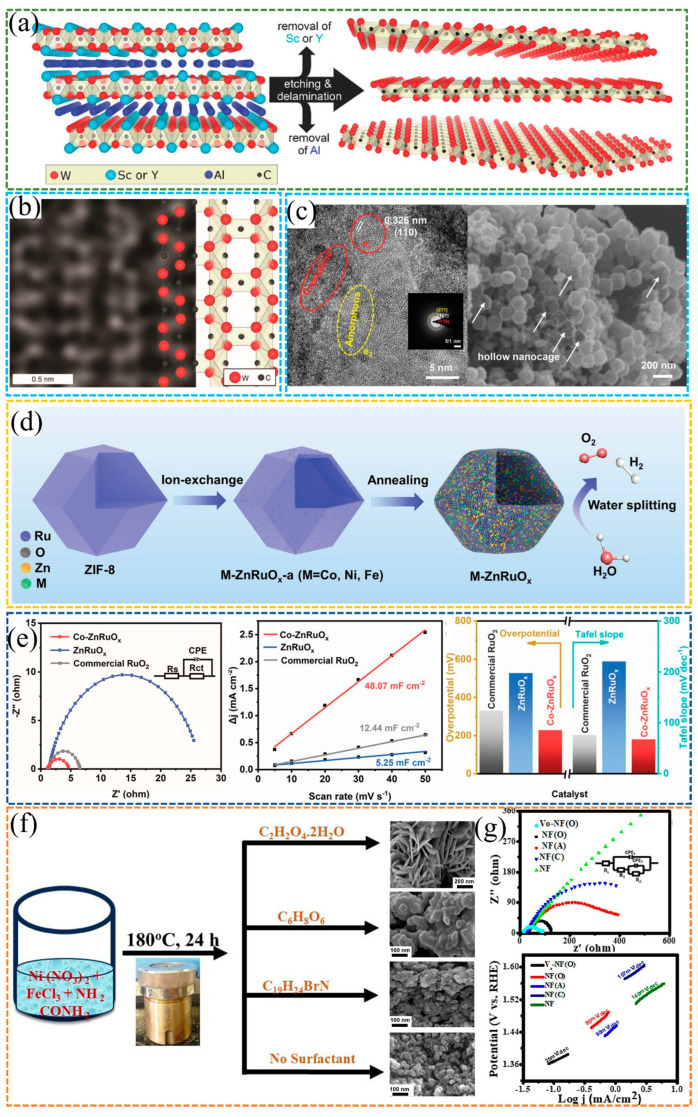
(**a**) Schematic diagram of W_1.33_C synthesis by etching and exfoliating (W_2/3_M_1/3_)_2_AlC (M = Sc or Y). (**b**) Image at atomic resolution with schematic atomic structure model for W_1.33_C [66]. Reproduced from Ref. [66] with permission. Copyright 2018 Wiley. (**c**) SEM image and HRTEM images of Co-ZnRuO_x_ nanocages. (**d**) Schematic illustration of the fabrication process of amorphous/crystalline M-ZnRuO_x_ nanocages. (**e**) OER EIS Nyquist plots and OER linear fitting of the capacitive current densities versus the scan rates of Co-ZnRuO_x_ nanocages, ZnRuO_x_ nanocages, and commercial RuO_2_, histogram of overpotentials at 10 mA·cm^−2^, and Tafel slopes of various catalysts [67]. Reproduced from Ref. [67] with permission. Copyright 2023 Wiley. (**f**) The schematic representation for the synthesis of NiFe_2_O_4_. (**g**) Nyquist plots and Tafel plots of all synthesized electrocatalysts [68]. Reproduced from Ref. [68] with permission. Copyright 2024 Wiley.

Rahele et al. [66] successfully synthesized W_1.33_C nanosheets using (W_2_/_3_M_1_/_3_)_2_AlC (M = Sc, Y) as the precursor through the concurrent removal of Al layers and interlayer Sc/Y atoms. The formation of ordered W vacancies, as visualized by SEM, not only enabled the successful fabrication of W-based MXenes but also remarkably enhanced their electrocatalytic property. Furthermore, compared with as-synthesized W_1.33_C counterparts, the annealed W_1.33_C nanosheets exhibited further improved electrocatalytic property, with a lower overpotential down to 320 mV at a current density of 10 mA·cm^−2^, as shown in Figure 8a,b.

As shown in Figure 8c–e, doping with metallic heteroatoms such as Co and Fe can remarkably enhance stability during the OER. Liu et al. [67] successfully constructed hollow M-ZnRuO_x_ nanocages by doping heteroatoms, including Co, Ni, and Fe, and combining with amorphous/crystalline heterostructures, which remarkably boosted the catalytic performance for both HER and OER in overall water splitting. Notably, Co-ZnRuO_x_ exhibited catalytic activity and stability that surpassed those of commercial Pt/C and RuO_2_. For OER, Co-ZnRuO_x_ delivered an overpotential of 224 mV and a Tafel slope of 67.55 mV·dec^−1^. EIS and C_dl_ measurements revealed faster charge transfer kinetics and a larger ECSA. Moreover, investigations assisted by DFT verified that Co doping not only modulated the electronic structure of Ru but also suppressed the overoxidation of Ru during OER, thus enabling the transition from a monofunctional to a bifunctional catalyst.

Senapati et al. [68] synthesized two-dimensional flower-like Ni-Fe oxide via oxalic acid-guided synthesis, with the material designated as NiF(O). As shown in Figure 8f,g, they further introduced oxygen vacancies through hydrazine hydrate reduction combined with ultrasonic treatment, successfully constructing oxygen vacancy-rich Ni-Fe oxide that was subsequently named V_O_-NiF(O). In terms of catalytic performance, V_O_-NiF(O) achieved an overpotential of only 260 mV at 10 mA·cm^−2^ and a Tafel slope of 25 mV·dec^−1^, a performance that surpasses most reported nickel–iron-based catalysts. EIS measurements and C_dl_ measurements demonstrated that V_O_-NiF(O) possessed faster charge transfer kinetics and a larger ECSA. To elucidate the underlying mechanism, X-ray Photoelectron Spectroscopy and Electron Spin Resonance analyses were conducted. These analyses confirmed that the presence of oxygen vacancies optimized the material’s electronic structure and facilitated the adsorption and desorption processes of reaction intermediates. Such modifications thereby remarkably enhanced the reaction kinetics. This study offers a novel vacancy engineering approach for the development of high-efficiency, stable non-precious metal catalysts for the OER.

Li et al. [69] proposed a novel, rapid, one-step molten salt-mediated phosphidation strategy to synthesize a heterostructure cobalt phosphide catalyst enriched with phosphorus vacancy defects. This strategy simplifies the synthesis workflow and facilitates the construction of high-performance bifunctional electrocatalysts, while the introduced phosphorus vacancies modulate the electronic structure of the catalysts. Consequently, the catalyst shows enhanced activity and stability toward overall water splitting under alkaline conditions. The DFT results indicate a positive correlation between vacancy concentration and predicted activity, and the approach is amenable to other transition metal phosphides.

As anionic defects, S [70], P [69], O [71], and B [72] vacancies can induce local electron redistribution and tailor the electronic structure. This electron redistribution drives electron transfer from vacancies to adjacent atoms, thereby endowing active sites with higher electron density. Furthermore, introducing vacancies creates additional electronic states near the Fermi level, which can elevate charge carrier concentration. This enhanced electronic conductivity accelerates charge transport and interfacial electron transfer, thereby increasing the apparent electrocatalytic rates. Furthermore, computational studies indicate that P vacancies can decrease the Gibbs free-energy change of key OER elementary steps, effectively lowering the theoretical overpotential by reducing the rate-determining free-energy barrier.

Within metal (oxy)hydroxides, lattice-level heteroatom substitution directly perturbs coordination and ligand fields, thereby reshaping adsorbate binding. Niu et al. [73] and Freibel et al. [48] demonstrate that selenium incorporation into FeOOH modulates the Fe coordination environment and charge distribution, lowers the free-energy barriers associated with oxygenated intermediates, and improves stability relative to undoped FeOOH. Taken together, these results support heteroatom doping—complementary to vacancy engineering—as a viable route to enhance intrinsic OER kinetics. When specific A-atom layers are removed from MAX phase precursors, even under mild conditions, adjacent atoms undergo inevitable detachment. This process gives rise to disordered anionic/cationic vacancies or vacancy clusters. Disordered atomic vacancies, such as oxygen vacancies and metal vacancies, have been observed in as-synthesized MXene nanosheets. These vacancies can increase the density of active sites, modulate the intrinsic electrical conductivity of MXenes, or tailor their surface electronic properties, which exert a significant influence on electrocatalytic performance [74,75].

Moving beyond isolated lattice substitution, placing single metal atoms adjacent to tailored anion defects can create cooperative active motifs and fast reconstruction pathways. Ru single atoms with S anions in NiFe-LDH promote charge transfer at both Ru and Ni centers, accelerate in situ conversion to NiFeOOH, and mitigate overoxidation at Ru, thereby narrowing the free-energy span and sustaining high-current operation [76]. This single-atom/anion co-doping constitutes a defect engineering lever that couples electronic micro-tuning with controlled reconstruction, complementing vacancy and heteroatom strategies above.

Heteroatom doping into metal or carbon materials has emerged as a prominent research focus in recent studies on electrocatalytic water splitting. During the doping process, heteroatoms introduced into the substrate simultaneously introduce hydrophilic moieties, including oxygen-containing and nitrogen-containing functional groups. These functional groups enhance the hydrophilicity of the catalyst surface, thereby promoting the adsorption and contact of electrolyte OH^−^ and reactant water molecules. Furthermore, co-doping with dual heteroatoms, with nitrogen (N) and O as typical examples, induces a synergistic effect. This synergistic effect further optimizes the electronic structure and surface properties of the catalyst, creating a more favorable catalytic environment than that achieved by single heteroatom doping. Additionally, the selection of different precursors enables the incorporation of distinct heteroatoms, which renders the experimental approach controllable and operationally feasible. Notably, heteroatoms such as nitrogen can serve as anchoring sites. They facilitate the formation of strong interactions, specifically Co-N bonds, with encapsulated metal nanoparticles like cobalt. This interaction prevents metal leaching, agglomeration, or oxidative deactivation during reactions, thereby enhancing the catalyst’s stability [77,78,79].

In a systematic investigation, Saha et al. [80] regulated heteroatom doping in metal organic gel-derived cobalt nanomaterials encapsulated within N and O dual-doped carbon onions. By tuning pyrolysis conditions, they achieved precise control of N and O contents at fixed cobalt loading. Higher N and O doping significantly enhanced OER activity and stability in alkaline media, surpassing Co–O benchmarks. Turnover frequency and mass activity analyses helped decouple surface area effects, supporting that increased heteroatom doping improves intrinsic activity. Moreover, the synthesis is simple and scalable, yielding catalysts with strong performance and stability, highlighting potential for practical application.

Pristine graphene is intrinsically inert in electrocatalytic processes, primarily because its delocalized π-conjugated network lacks favorable binding sites for reaction species. To address this limitation, electrocatalytic activity can be endowed to graphene by incorporating non-carbon heteroatoms such as B, S, N, and P. Heteroatoms like N, due to their distinct electronegativities from carbon, can modulate the charge distribution within the carbon framework. This modulation induces adjacent carbon atoms to carry positive charges, which then serve as active sites for the adsorption of reaction intermediates, including *OH, *O, and *OOH. Notably, heteroatom doping, with nitrogen doping as a prominent example, can introduce defects and additional charge carriers into carbon materials. These changes enhance the electrical conductivity of carbon support and facilitate electron transfer during electrocatalytic reactions. Additionally, heteroatoms can alter the electronic states of the conjugated carbon framework by integrating heterogeneous catalytic phases [81,82,83,84].

Lu et al. [26] synthesized NCNTs with high graphitic N content by chemical vapor deposition from acetonitrile at low temperatures. The NCNTs showed HER activity, higher than Pt/C but slightly lower than nickel foam, and they also exhibited strong OER activity. Density functional theory indicated that the enhanced OER activity originates primarily from the heteroatoms in the graphitic N-rich NCNTs rather than from any synergistic effect with the nickel foam substrate. These results suggest practical guidelines for designing carbon-based catalysts that are effective for both HER and OER.

Taken together, N-, P-, S-, and B-doped carbon nanomaterials introduce multiple potential active sites and tune band gaps, densities of states, and local charge distribution. This electronic modulation governs charge transfer energetics and the stabilization of key intermediates, which can lower overpotentials and increase rates [17]. Specific dopant arrangements may also enable more favorable reaction pathways. As a result, appropriately engineered dopants and defect structures can synergistically improve the bifunctional performance of carbon catalysts for HER and OER.

Tang et al. [85] proposed a theory-guided strategy that markedly elevates oxygen-electrocatalysis on carbon by introducing high-entropy heteroatoms. Lowering local symmetry in graphitic carbon perturbs the π-electron network and avoids both excessively strong and excessively weak binding of oxygenated intermediates in ORR and OER. The resulting catalyst, which embeds five single-atom metals (Fe, Mn, Co, Ni, and Cu) together with two nitrogen sources, delivered exceptional bifunctional activity in alkaline media and outperformed commercial Pt/C and RuO_2_ benchmarks for ORR and OER, respectively.

### 4.3. Phase and Interface Engineering

Operando studies established in Section 4.1 that Ni–Fe precursors reconstruct under anodic bias into β- or γ-type NiOOH. Here, we focus on phase and interface levers that work in concert with this reconstructed working state. Interface coupling provides a second and complementary route. Conversion of a Ru-coupled Ni–MOF into sheet-like porous RuO_2_–NiO produces a heterostructure in which the RuO_2_ and NiO interface downshifts the d band center, weakens adsorption of oxygenated intermediates, and accelerates water dissociation, yielding low overpotential at 10 mA·cm^−2^ with good stability [86].

Mesoscale field management and microenvironment design further raise the rate ceiling under device-relevant operation. An engineered interfacial layer enriches local hydroxide concentration, lowers interfacial resistance, improves mass transport, and extends durability in zero-gap anion exchange membrane cells compared with unmodified electrodes [76].

A wide range of precursors, including phosphides, sulfides, selenides, nitrides, and borides, reconstruct in alkaline OER to Ni–Fe(oxy)hydroxides. In these systems, the composition and architecture before reconstruction primarily govern charge transport, accessible site density, and bubble management, while the skin that forms under bias provides the active motif. Spinel and perovskite oxides also develop (oxy)hydroxide skins. When evaluated at device-relevant current densities, they function as reconstructable backbones or robust scaffolds whose interfaces and vacancies can be engineered to add activity [87].

**Figure 9:**
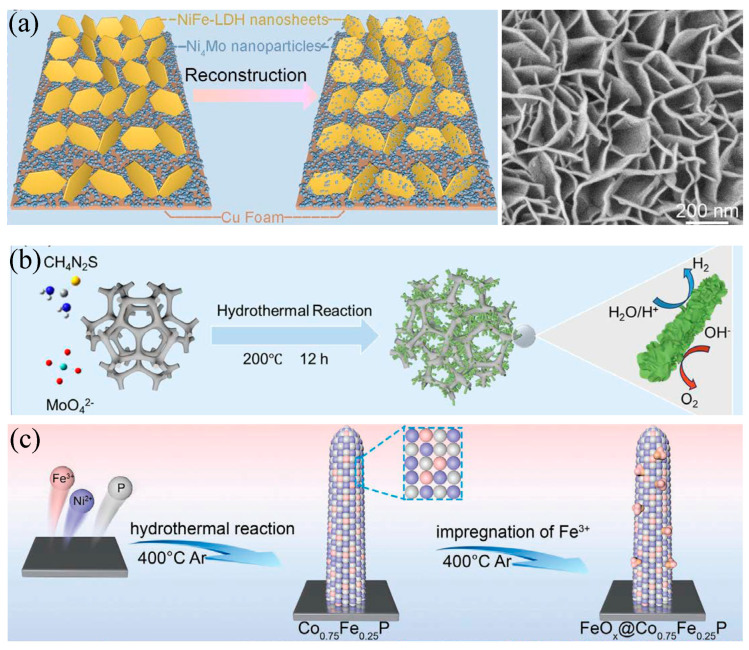
Phase and interface engineering for alkaline water splitting. (**a**) Operando reconstruction of NiFe LDH nanosheets on Ni Mo nanoparticles supported by Cu foam improves durability and activity [88]. Reproduced from Ref. [88] with permission. Copyright 2024 Wiley. (**b**) Hydrothermal synthesis of vacancy-rich MoS_2_ combined with crystalline Ni_3_S_2_ forms a conductive heterointerface that accelerates charge transfer and water dissociation [90]. Reproduced from Ref. [90] with permission. Copyright 2025 Wiley. (**c**) Interface construction through growth of Co_0.75_Fe_0.25_P nanorods followed by Fe^3+^ impregnation and phosphidation yields FeO_x_@Co_0.75_Fe_0.25_P with abundant active sites and robust performance [89]. Reproduced from Ref. [89] with permission. Copyright 2024 Wiley.

Rational control of phase and interfaces provides unified levers to activate alkaline OER catalysts. Phase engineering through amorphization and through coupling crystalline and amorphous domains relaxes local coordination, speeds the in situ transformation into the working oxyhydroxide, and improves durability under bias [88,89]. Lattice oxygen regeneration can be promoted in NiFe LDH by engineering lattice oxygen regeneration, which sustains activity at high current and extends operational lifetime [90], as shown in Figure 9.

Interface engineering creates built-in fields and accelerates interfacial charge transfer and water dissociation, which lowers interfacial barriers for *OH formation and gas release. In perovskite composites, cation defects induce in situ phase separation. The resulting biphasic interface provides coherent pathways for electrons and oxide ions, increases oxygen vacancy mobility, and strengthens the coupling between oxygen 2p states and metal-d states. Under operating bias, this architecture promotes lattice oxygen participation and accelerates O–O bond formation while preserving rapid vacancy healing. The heterointerface creates an internal field that assists charge separation and lowers interfacial barriers for water activation. It also anchors surface cations and mitigates A-site and B-site leaching, which slows phase drift during long-term electrolysis [91]. Amorphous MoS_2_ combined with crystalline Ni_3_S_2_ shows that vacancy-rich sulfide interfaces lower adsorption-free energies and enable bifunctional overall water splitting at low cell voltage [90]. Interfacial field effect enhancement of non-Faradaic processes explains why optimized junctions outperform single-phase counterparts [89]. Defect-rich Fe_3_O_4_ precatalysts stabilized by Co and S atomic pair co-doping link interfacial rearrangement to higher accessible site density and robust OER kinetics [92]. Copper-doped nickel architectures, although developed for HER, illustrate a transferable blueprint in which dopant-induced electronic tuning works together with nanoscale interfaces to boost catalytic turnover, and this paradigm applies directly to alkaline OER design [93]. Bismuth incorporated into nickel oxyhydroxides tailors electronic structure and hydrophilicity, facilitates intermediate transport and charge transfer at the electrolyte and catalyst boundary, and delivers superior water oxidation performance under alkaline conditions [94].

Table 1 summarizes representative catalyst performance for alkaline OER. The reported metrics include iR-corrected overpotential at a fixed current density, Tafel slope, and durability under sustained load. A vertical comparison shows that multiple systems already operate stably near 0.1–1 A·cm^−2^, while gaps remain in achieving both higher current densities and longer lifetimes. These gaps point to targeted opportunities for deeper optimization in reconstruction control, defect engineering, and phase–interface coupling.

Taken together, a coherent design space emerges for alkaline OER. First, control and accelerate reconstruction to rapidly form the active (oxy)hydroxide phase. Second, apply heteroatom doping and electronic micro-tuning to refine the energetics of hydroxyl, oxygen, and peroxide intermediates and to improve charge transport. Third, engineer interfaces and microenvironments, from nano and atomic field confinement to tailored membrane–electrode layers that enrich hydroxide and ease intermediate desorption. Fourth, stabilize the composition by managing ion migration to suppress metal dissolution and lock the reconstructed phase. When these levers are combined and validated under device-relevant conditions, concurrent gains in activity and durability can be realized.

MOF provides a programmable platform for phase and interface design. Its tunable metal node and organic linker allow the precursor to preset coordination environments and pore microenvironments during synthesis, and mild post-treatments can guide conversion into conductive skeletons and purposeful heterointerfaces [95,96]. Graphene-confined MOFs and related conductive architectures address conductivity and mass transport limits while preserving MOF-derived active motifs under alkaline OER [95]. The Ru–Ni MOF to RuO_2_–NiO example illustrates how MOF-derived scaffolds can deliver phase control and interface control in one route, which aligns with Wiley reviews that map MOF design, conversion, and interface strategies for water splitting [97].

## 5. Summary and Outlook

Alkaline water electrolysis powered by renewable electricity is a leading route to green hydrogen and a practical pathway toward carbon neutrality. Progress is constrained by the sluggish kinetics of the OER in alkaline media, which limits device efficiency and durability. Advancing highly active and stable OER catalysts, therefore, requires a deeper, mechanistically grounded understanding of reaction pathways and kinetics under realistic operating conditions [1].

This review summarizes OER fundamentals in alkaline media, highlights key electrochemical parameters, and discusses AEM and LOM. We then survey structure–activity strategies that tune active sites and reaction environments, including defect and vacancy engineering, heterostructure design, heteroatom doping, morphology control, and particle size effects. These approaches, when combined with rigorous testing and reporting practices, provide a route to optimize activity and stability [21,40].

Mechanistic Understanding

Despite substantial advances, important uncertainties remain. Discrepancies often originate from variations in electrolytes, potentials, and operando reconstruction during operation. The commonly invoked AEM and LOM do not capture all possible pathways, and additional effects such as interfacial water structure, local electric fields, and specific adsorption by electrolyte ions can influence both kinetics and stability. Continued development of operando diagnostics together with quantitative microkinetic modeling is essential to assign mechanisms reliably and to connect measured activity to elementary steps. At device-relevant temperatures and current densities, mechanism assignment should explicitly consider dual site cooperation and lattice oxygen participation to avoid misattributing apparent kinetics [36,45,58].

High Activity and Stability

A central challenge is to achieve high intrinsic activity together with long lifetimes at technologically relevant current densities. Many state-of-the-art systems excel in one dimension but not the other. Promising directions include electronic structure tuning that balances intermediate binding, interface designs that facilitate charge and mass transport, and supports that resist corrosion and delamination. Community benchmarks should include iR-corrected overpotential at fixed current density, ECSA or turnover frequency, time at current stress testing at 100 to 500 mA·cm^−2^ or higher, and stability metrics that quantify metal loss. We recommend reporting overpotential no higher than 350 to 400 mV at 500 mA·cm^−2^ for at least 100 to 1000 h and full cell voltages in the range of 1.8 to 1.9 V at 1 A·cm^−2^ in zero-gap alkaline devices [54,62,68].

Bifunctional Catalysts for Overall Water Splitting

For practical devices, bifunctional electrodes can simplify stack design and reduce costs. In this review, we keep an OER-centric focus and evaluate bifunctional systems primarily through their OER-relevant metrics. Heterostructures that combine complementary phases enable charge redistribution and dual site pathways, and they often reconstruct to active oxyhydroxides under bias. Evaluation should prioritize full cell performance in zero-gap architectures with realistic compression and flow, since electrode porosity, wettability, and bubble management can dominate performance beyond 100 mA·cm^−2^ [67,91].

In Situ Characterization and Theoretical Calculations

Identifying true active centers in complex and evolving materials remains difficult, especially for multicomponent and high-entropy systems. Progress will depend on operando spectroscopy and microscopy that track phase evolution and oxidation states, together with theory that includes explicit solvation, applied potential, electrolyte cations and anions, and interfacial electric fields. Data-driven screening can accelerate discovery when coupled with stability constraints and reconstruction-aware models [30,58].

Outlook

Evidence from operando studies shows that many Ni- and Fe-containing precatalysts reconstruct into β- or γ-type NiOOH skins under anodic bias and that accelerating the induction to this working state while suppressing metal loss is central to durable, high-rate OER. Priority should be given to reconstruction rate control, dopant-guided phase locking, and ion migration management, with durability verified at ≥100–500 mA·cm^−2^ for 100–1000 h and minimal drift in η and R_ct_ in zero-gap cells [48,53,54,55,56,61].

Mechanism assignments must be made under operating conditions. Combining ^18^O labeling and complementary operando spectroscopies with constant potential, explicitly solvated modeling can discriminate AEM from LOM, quantify crossovers, and link oxygen redox to kinetics. These tools should be applied at the intended operating temperature and current density so that lattice oxygen participation and dual site cooperation are not misattributed [28,42,43].

Phase and interface engineering provides a practical route to relax scaling constraints and raise the transport ceiling. Perovskite composites created through cation defect-induced in situ phase separation build strongly coupled biphasic interfaces that enable fast electron and oxygen vacancy transport while preserving vacancy healing. These junctions lower interfacial barriers for water activation and gas release and maintain composition against A-site and B-site leaching, yielding lower iR-corrected overpotentials and sustained operation at device-relevant current densities [86,88,89,90,91].

Temperature is a design variable. Elevated temperature perovskite studies at approximately 50–80 °C demonstrate increased lattice oxygen participation and higher apparent rates due to enhanced vacancy mobility and stronger O-2p to metal-d hybridization, but they also highlight risks of cation leaching and phase drift. Future evaluations should, therefore, pair high temperature operation with vacancy healing and cation retention strategies and report stability and mechanism at the target temperature in zero-gap architectures [53].

Electrolyte engineering deserves attention equal to solid-state tuning. Trace Fe control, carbonate management, and cation and anion identity shift kinetics by amounts comparable to doping. Reports should include electrolyte composition and impurity budgets, Faradaic efficiency, and solution phase metal loss together with solid-state post-mortem analysis, especially above 100 mA·cm^−2^ [53,54,88,89,90,94].

## Figures and Tables

**Table 1:** Representative alkaline OER performance in 1.0 M KOH (a–e indicate the current density j used for overpotential reporting: ^a^ 10 mA·cm^−2^, ^b^ 20 mA·cm^−2^, ^c^ 100 mA·cm^−2^, ^d^ 500 mA·cm^−2^, ^e^ 1000 mA·cm^−2^).

	Materials	Overpotential /mV	Tafel Slope /mV·dec^−1^	Stability /h
Section 4.1	NixCryO	^a^ 270	50	^c^ 475 [54]
	Cu_2_S/CoFeCuOOH	^a^ 170, ^c^ 258	41	^c^ 100 [57]
	Mn SA-CoP NNs	^c^ 189	69.4	^c^ 100 [59]
	S-NiFeZn LDH/NiF	^a^ 201, ^c^ 235	44.2	^e^ 400 [60]
	CuS	^a^ 192	144	^a^ 120 [61]
Section 4.2	Co-ZnRuO_x_	^a^ 224	67.55	^b^ 120 [67]
	V_o_-NiF(O)	^a^ 260	25	^a^ 18 [68]
	CoP_x_-350	^a^ 351	91	^d^ 24 [69]
	z-NiFe	^e^ 190	28.5	^e^ 14,000 [76]
	Ru/RuO_2_-NbB_2_-v	^a^ 270	42	^a^ 20 [72]
Section 4.3	NiFe-LDH/Ni_4_Mo	^a^ 20.9, ^c^ 115.2	42	^c^ 150 [88]
	MoS_2_@Ni_3_S_2_-NiF	^a^ 245	25	^c^ 100 [90]
	FeO_x_@Co_0.75_Fe_0.25_P	^c^ 240	35.1	^e^ 120 [89]
	Co-FeOOH-Ov/IF	^c^ 296	43.3	^e^ 200 [92]
	Ni97Bi_3_(OH)_x_	^a^ 248, ^e^ 394	33.3	^d^ 1000 [94]

## Data Availability

No new data were created or analyzed in this study. Data sharing is not applicable to this article.
